# Two-Dimensional *N*-Glycan Distribution Mapping of Hepatocellular Carcinoma Tissues by MALDI-Imaging Mass Spectrometry

**DOI:** 10.3390/biom5042554

**Published:** 2015-10-15

**Authors:** Thomas W. Powers, Stephanie Holst, Manfred Wuhrer, Anand S. Mehta, Richard R. Drake

**Affiliations:** 1 Department of Cell and Molecular Pharmacology and Experimental Therapeutics and MUSC Proteomics Center, Medical University of South Carolina, 173 Ashley Avenue, Charleston, SC 29425, USA; E-Mail: ThomasWesley.Powers@pfizer.com; 2 Center for Proteomics and Metabolomics, Leiden University Medical Center, Leiden 2333ZA, The Netherlands; E-Mails: s.holst@lumc.nl (S.H.); m.wuhrer@lumc.nl (M.W.); 3 Division of BioAnalytical Chemistry, VU University, Amsterdam 1081HV, The Netherlands; 4 Department of Molecular Cell Biology and Immunology, VU University Medical Center, Amsterdam 1007MB, The Netherlands; 5 Department of Microbiology and Immunology, College of Medicine, Drexel University, 2900 W. Queen Lane, Philadelphia, PA 19129, USA; E-Mail: anand.mehta@drexelmed.edu

**Keywords:** *N*-linked glycosylation, formalin-fixed paraffin-embedded tissue, hepatocellular carcinoma, MALDI imaging mass spectrometry, glycoprotein

## Abstract

A new mass spectrometry imaging approach to simultaneously map the two-dimensional distribution of *N*-glycans in tissues has been recently developed. The method uses Matrix Assisted Laser Desorption Ionization Imaging Mass Spectrometry (MALDI-IMS) to spatially profile the location and distribution of multiple *N*-linked glycan species released by peptide *N*-glycosidase F in frozen or formalin-fixed tissues. Multiple formalin-fixed human hepatocellular carcinoma tissues were evaluated with this method, resulting in a panel of over 30 *N*-glycans detected. An ethylation reaction of extracted *N*-glycans released from adjacent slides was done to stabilize sialic acid containing glycans, and these structures were compared to *N*-glycans detected directly from tissue profiling. In addition, the distribution of singly fucosylated *N*-glycans detected in tumor tissue microarray cores were compared to the histochemistry staining pattern of a core fucose binding lectin. As this MALDI-IMS workflow has the potential to be applied to any formalin-fixed tissue block or tissue microarray, the advantages and limitations of the technique in context with other glycomic methods are also summarized.

## 1. Introduction

Hepatocellular carcinoma (HCC), a subtype of liver cancer that accounts for approximately two-thirds of all liver cancers, is among the most common and aggressive malignancies worldwide [1,2]. Early detection disease biomarkers are highly sought after and offer the potential to drastically improve patient outcome. Both proteomic and glycomic approaches have led to significant advances in HCC biomarker research [2]. Glycoproteins such as peroxiredoxin 3 [3,4], osteopontin [5,6,7], and alpha fetoprotein (AFP) [8] have been identified as potential HCC biomarkers. However, detection of these proteins alone display limited sensitivity in the background of liver cirrhosis or benign diseases. It has been observed that α-1,6 core fucosylated AFP offers improved specificity for HCC than AFP alone [8,9,10]. Currently, core fucosylated AFP assessed through the AFP-L3 test, is the only test approved by the United States Food and Drug Administration for the detection of hepatocellular carcinoma. Elevated levels of core fucosylation are not specific for AFP, but are also observed on transferrin, alpha-1-antitrypsin, GP73 and other proteins [11,12,13,14,15]. Total serum glycomic studies have also identified increases in core α-1,6 linked and α-1,3 linked outer-arm fucosylation, *N*-glycan branching, and sialylation in HCC samples [16,17,18,19].

We have recently developed a matrix-assisted laser desorption-ionization imaging mass spectrometry (MALDI-IMS) approach for profiling *N*-glycans directly in frozen and formalin-fixed paraffin-embedded (FFPE) tissues [20,21]. The method relies on the spraying of a molecular coating of peptide *N*-glycosidase F (PNGaseF) to release *N*-glycans directly on tissues mounted on glass slides prior to matrix application. Relative to other classes of biomolecules targeted by MALDI-IMS, the method offers ready identification of the individual glycan species and creation of structural reference databases [21]. In this report, the utility of the method is demonstrated for profiling multiple *N*-glycans using different FFPE tissue slices of HCC. Current data analysis approaches are also summarized, as well as use of a novel derivatization method to stabilize sialic acids in a linkage-dependent/differentiating manner, and therefore better characterize larger, sialic acid containing *N*-glycans [22]. As the methodology is still evolving, areas to improve glycan detection and structural characterization by MALDI-IMS are discussed, as well as the context of how other glycan analysis approaches complement the method. As nearly all current cancer biomarkers are glycoproteins or carbohydrate antigens [23,24], a global analysis of *N*-glycans from HCC tissue sections using MALDI-IMS could extend and complement the continued development of *N*-glycan biomarkers associated with HCC.

## 2. Materials and Methods

### 2.1. Materials

Trifluoroacetic acid, α-cyano-4-hydroxycinnamic acid (CHCA), sodium hydroxide (NaOH), 1-hydroxybenzotriazole hydrate (HOBt), and trypsin were obtained from Sigma-Aldrich (St. Louis, MO, USA). Ammonium bicarbonate, HPLC grade methanol, ethanol, acetonitrile, xylene and water were obtained from Fisher Scientific (Pittsburgh, PA, USA). 1-(3-Dimethylaminopropyl)-3-ethylcarbodiimide hydrochloride (EDC) was obtained from Oakwood Chemical (West Columbia, SC, USA). Tissue Tack positively charged microscope slides were purchased from Polysciences, Inc. (Warrington, PA, USA). Citraconic anhydride for antigen retrieval was from Thermo Scientific (Bellefonte, PA, USA). Recombinant Peptide *N*-Glycosidase F (PNGaseF) from *Flavobacterium* *meningosepticum* was expressed and purified as previously described [20], and is available commercially as PNGase F Prime™ from Bulldog Bio (Portsmouth, NH, USA).

### 2.2. FFPE Tissues

All but one tissue sections used in this study were de-identified and obtained commercially from Biochain (Newark, CA, USA). These include a slide with two FFPE tissue sections from a 60 year old female with a poorly differentiated hepatocellular carcinoma; patient matched tumor and normal FFPE tissue sections from a 53 year old male with hepatocellular carcinoma, and a patient matched renal cell carcinoma tumor and lymph node tissue with renal cell carcinoma metastasis from a 28 year old female. A subset of a commercially available renal tissue microarray (TMA) from Biochain was also analyzed, where two cores from each patient (oriented vertically) were present. One de-identified pancreas tissue was obtained by MUSC and was determined to be not human research classifications by the respective Institutional Review Boards at MUSC.

### 2.3. Washes for Deparaffinization and Rehydration

Slide preparation proceeded as described in our previous paper [21]. Briefly, FFPE tissue sections not acquired precut from Biochain, were sectioned at 5 μm and mounted on positively charged slides compatible with the Bruker slide adaptor. Standard ITO coated slides used for most MALDI imaging studies do not need to be used with this FTICR-MALDI configuration. All slides were heated at 60 °C for 1 h to ensure tissue adhesion to the slide. After cooling, the slide was deparaffinized by washing with xylene and rehydrated in a series of ethanol and water washes. Citraconic anhydride (Thermo) was used as the antigen retrieval buffer and the retrieval process took place over 25 min in a vegetable steamer. After allowing the buffer to cool, the buffer was gradually exchanged to 100% water. The slide was then desiccated to dryness prior to enzymatic digestion.

### 2.4. N-Glycan MALDI-IMS

An ImagePrep spray station (Bruker Daltonics, Billerica, MA, USA) was used to coat the slide with a 0.2 mL aqueous solution of PNGaseF (20 µg total/slide) as previously described [21]. As negative control, adjacent control tissue slices were shielded from PNGaseF application by covering the tissue section with a glass slide. Digestion occurred in a humidified chamber at 37 °C for 2 h. Slides were desiccated prior to α-cyano-4-hydroxycinnamic acid matrix application (0.021 g CHCA in 3 mL 50% acetonitrile/50% water and 12 µL 25% TFA) using the ImagePrep sprayer. Released glycan ions were detected using a Solarix dual source 7T FTICR mass spectrometer (Bruker Daltonics) (*m*/*z* 690–5000) with a SmartBeam II laser operating at 1000 Hz, a laser spot size of 25 μm. Following MS analysis, data was loaded into FlexImaging Software focusing on the range *m*/*z* = 1000–4000 and reduced to 0.95 ICR Reduction Noise Threshold. FlexImaging 4.0 (Bruker Daltonics) was used to generate images of differentially expressed glycans. Observed glycans were searched against the glycan database generated using GlycoWorkbench [25]. Presented glycan structures were generated in GlycoWorkbench and represent putative structures determined by combinations of accurate *m*/*z* and off-slide derivatization experiments. CASI/CID was done as previously described [20,21].

### 2.5. Ethyl Esterification

*N*-glycans were extracted from slides as described previously and dried by vacuum centrifugation [20]. The ethyl esterification protocol, including the modification and enrichment, was adapted from Reiding *et* *al*. [22]. Briefly, 2 µL water and 40 µL 0.25 M HOBt/EDC were added to dried glycans followed by incubation at 37 °C for 1 h. 40 µL acetonitrile was added and the mixture was placed at −20 °C for 20 min. Glycans were enriched using cotton-HILIC tips according to Selman *et* *al*. [26]. Briefly, cotton wool composed of 100% cotton (Assured, Rio Rancho, NM, USA) was inserted in 20 µL tips and equilibrated with 10 µL water three times followed by 10 µL 85% ACN three times. Samples were loaded and unbound material removed by washing three times with 10 µL 85% acetonitrile with and without 1% TFA, respectively. Tip-bound glycans were eluted in 10 µL water. Enriched and modified glycans were spotted on an Anchorchip MALDI plate (Bruker Daltonics) with 2,5-dihydroxybenzoic acid (DHB) at a concentration of 5 mg/mL in 50% ACN/50% water/1 mM NaOH. Ethanol was then used for recrystallization.

### 2.6. Lectin Histochemistry

An HCC TMA slide (Catalog No.: Z7020059, Lot No.: B506168) was purchased from Biochain, Inc. This array consisted of 16 cases of liver cancer, each in duplicate, with corresponding uninvolved tissue from the same patient acting as controls. All the tissues were from surgical resection. Patients had a mean age of 47.56 years (range of 33–68 years) with a 8:1 ratio of males to females. Tissue slides were deparaffinized by using PROTOCOL SafeClear II clearing agent (Fisher Scientific), followed by rehydration though a series of graded ethanol. Lectin histochemistry was performed at room temperature, unless otherwise indicated. Endogenous peroxidase activity was blocked using 3% hydrogen peroxide, followed by heat-mediated antigen retrieval using Universal Antigen Retrieval Reagent (R & D Systems, Minneapolis, MN, USA). Tissues were fixed with 4% formaldehyde solution followed by permeabilization with 0.5% IGEPAL. Prior to staining, the TMA slides were blocked using serum-free Dako Protein Block (Carpinteria, CA, USA). Next, the detection of core fucosylation was conducted using biotinylated recombinant N224Q rAAL lectin [27], diluted in Dako High Background Reducing Diluent solution to make the working final concentration of 500 ng/mL. Bound, biotinylated lectin was detected using streptavidin horseradish peroxidase (Vector Laboratory, Burlingame, CA, USA), and color was developed using 3,3'-diaminobenzidine (DAB) Chromogen from Dako (Carpinteria, CA, USA). Slides were visualized using an E200 Binocular Compound Biological Microscope from Nikon Instrument (Melville, NY, USA) and an IX71 Inverted Microscope from Olympus Imaging America, Inc. (Center Valley, PA, USA).

## 3. Results

### 3.1. Influence of Histopathology on MALDI-IMS of N-Glycans

**Figure 1:**
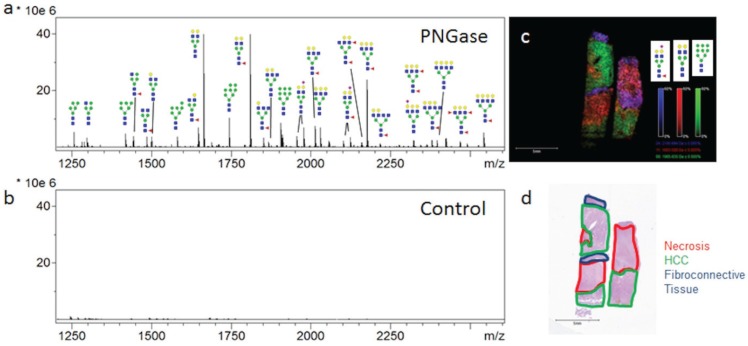
PNGaseF Releases *N*-linked Glycans from HCC Tissue in a Spatially Defined Manner. A HCC tissue slide (Biochain) containing two serial slices of HCC tissue was analyzed by *N*-glycan MALDI-IMS (**a**); One section was shielded from enzyme application to serve as a negative control (**b**); Three representative ions were selected in FlexImaging and displayed as an overlay image (**c**). Hex5dHex1HexNAc4NeuAc (*m*/*z* = 2100.759, blue), Hex5HexNAc4 (*m*/*z* = 1663.582, red) and Hex9HexNAc2 (*m*/*z* = 1905.612, green) each display unique localization within the tissue slice (**c**); As highlighted in the H & E stain of the slide (**d**), indicated regions were identified to be fibroconnective tissue (blue), necrotic tissue (red), and HCC tissue (green). Tri- and Tetra-antennary compositions are shown with representative glycan structures. Glycan structures with single fucoses are shown as being attached to the core *N*-acetylglucosamine for consistency. All of these representations need to be confirmed by other methods.

The ability to profile and determine the two-dimensional localization and histopathology of multiple *N*-glycans in tissues has been developed using a MALDI-IMS approach [20,21]. The goal of the studies were to present new data for analysis of *N*-glycan distribution in different FFPE hepatocellular carcinoma tissues, in order to highlight the capabilities and limitations of the method, how it compares with other glycan analysis approaches, and identify areas of improvement. Initial MALDI-IMS of a commercially available FFPE HCC tissue of complex histopathology demonstrated the specific release of *N*-glycans following PNGaseF digestion and the ability of released glycans to distinguish tissue subtypes and pathologies. Two serial sections were prepared identically with the exception that one tissue received PNGaseF while the other was shielded from enzyme application to serve as a negative control. Average spectra of each tissue region revealed a robust signal increase following PNGaseF digestion (Figure 1a) compared to the control tissue (Figure 1b). Putative glycan structures were annotated using GlycoWorkbench [25] based on mass accuracy and previous studies [20,21], but no information is available regarding specific anomeric linkages. All glycan structures reported herein are the [M + Na]^+^ adducts unless otherwise noted, and a representative list of detected *N*-glycan compositions are in Table 1. Three representative glycans, Hex5dHex1HexNAc4NeuAc1 (*m*/*z* 2100.759, blue), Hex5HexNAc4 (*m*/*z* 1663.582, red) and Hex9HexNAc2 (*m*/*z* 1905.612, green) were selected and shown in an overlay image (Figure 1c). The three glycans map to the tissue histopathology marked on the H & E stain (Figure 1d), withthree evident tissue morphologies; necrosis (outlined in red), HCC tissue (outlined in green) and fibroconnective tissue (outlined in blue). The three *N*-glycans in Figure 1c were selected to illustrate the regiospecific distribution of them in relation to the different histopathologies. Images of other *N*-glycans localized to the three regions are shown in Supplemental Figure 1. Interestingly, high mannose glycans were elevated in the tumor tissue, while fucosylated or sialylated/fucosylated complex diantennary glycans were elevated in the fibroconnective tissue.

**Figure 2:**
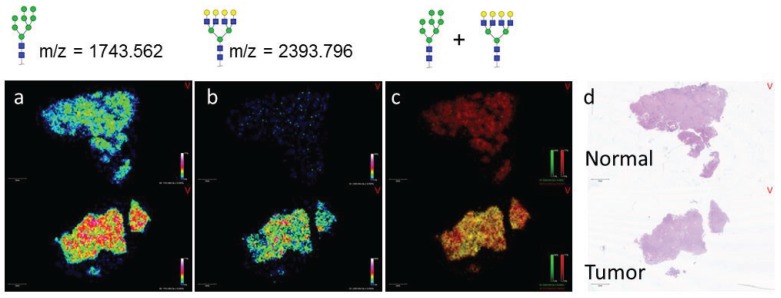
MALDI-IMS Reveals *N*-glycans that can Distinguish Tumor from Normal in Matched HCC Tissue Samples. HCC and adjacent normal tissues from the same donor (Biochain) were analyzed by MALDI-IMS after PNGaseF release of *N*-glycans. *m*/*z* = 1743.562 (Hex8HexNAc2 + Na) was present in both tissues (**a**); while *m*/*z* = 2393.796 (Hex7HexNAc6 + Na) was primarily in the tumor tissue only (**b**); An overlay view of these two glycan species where *m*/*z* = 1743.562 is displayed in red and *m*/*z* = 2393.796 is displayed in green confirms this finding. In the overlay, the normal tissue is primarily red but the tumor tissue is yellow in color as a result of both glycan species being present (**c**); H & E staining of both tissues (**d**).

While the initial experiment demonstrated the ability of *N*-glycan localization to define pathology in HCC tissue blocks, the ability to distinguish tumor *vs*. normal tissue is of more clinical significance. Analysis of a patient matched tumor and normal FFPE tissue sample revealed overall glycan heterogeneity between the two tissues. While Hex8HexNAc2 (*m*/*z* 1743.565, red) is present in both the normal and tumor sections, Hex7HexNAc6 (*m*/*z* 2393.854, green) is largely absent in the normal tissue (Figure 2a,b). This observation is evident in the image overlay, where the normal tissue image is red in color due to the presence of Hex8HexNAc2 and the absence of Hex7HexNAc6, while the tumor tissue is yellow due to the presence of both Hex7HexNAc6 and Hex8HexNAc2 (Figure 2c,d). This finding is consistent with our previous studies made in the analysis of an HCC TMA. Interestingly, while Hex8HexNAc2 is present in both the matched tumor and normal tissues and was elevated in the normal HCC tissue (Figure 2a), it is elevated in the tumor tissue compared to necrotic and fibroconnective tissue regions (Supplemental Figure S1). This trend emphasizes the importance of coupling histological analysis with the MALDI-IMS technique.

**Table 1:** Comparative Analysis of *N*-glycans from MALDI-IMS and Ethyl Esterification. *N*-glycans from an HCC tumor tissue were extracted as described for Figure 4. Theoretical glycan *m*/*z* values were generated using GlycoWorkbench and are displayed as the [M + Na]^+^ value. The *m*/*z* values of the *N*-glycans from MALDI-IMS and following the ethyl esterification protocol are also provided as the [M + Na]^+^ value, unless otherwise stated. *N*-glycans bearing a single sialic acid residue are ^a^ lactonized or ^b^ ethylated. In the case where one glycan has two sialic acid residues, the glycan has ^c^ one lactonized and one ethylated sialic acid or ^d^ two ethylated sialic acids. Sialylated glycans from MALDI-IMS are present as both the [M + Na]^+^ and ^e^ [M + 2Na]^+^ adduct.

		MALDI-IMS		Ethyl Esterification	
Observed Glycan	Database m/z	Observed	*m/z*	Observed	*m/z*
Hex5HexNAc2	1257.422	Yes	1257.497	Yes	1257.422
Hex4HexNAc3	1298.449	Yes	1298.522	Yes	1298.450
Hex6HexNAc2	1419.476	Yes	1419.530	Yes	1419.473
Hex4dHex1HexNAc3	1444.507	Yes	1444.546	Yes	1444.507
Hex3dHexHexNAc4	1485.534	Yes	1485.587	Yes	1485.540
Hex4HexNAc4	1501.529	Yes	1501.592	Yes	1501.536
Hex7HexNAc2	1581.528	Yes	1581.562	Yes	1581.532
Hex4dHex1HexNAc4	1647.587	Yes	1647.617	Yes	1647.589
Hex5HexNAc4	1663.581	Yes	1663.582	Yes	1663.591
Hex8HexNAc2	1743.581	Yes	1743.562	Yes	1743.592
Hex5dHex1HexNAc4	1809.639	Yes	1809.661	Yes	1809.644
Hex4dHex1HexNAc5	1850.666	Yes	1850.686	Yes	1850.683
Hex5HexNAc5	1866.661	Yes	1866.663	Yes	1866.686
Hex9HexNAc2	1905.634	Yes	1905.612	Yes	1905.647
Hex5HexNAc4NeuAc1	1954.677	Yes	1954.589	Yes	1936.692 ^a^
			1976.707 ^e^		1982.733 ^b^
Hex5dHex2HexNAc4	1955.697	Yes	1955.692	Yes	1955.719
Hex5dHex1HexNAc5	2012.719	Yes	2012.711	Yes	2012.745
Hex6HexNAc5	2028.714	Yes	2028.737	Yes	2028.737
Hex5dHex1HexNAc4NeuAc1	2100.735	Yes	2100.759	Yes	2082.733 ^a^
			2122.720 ^e^		2128.798 ^b^
Hex5dHex2HexNAc5	2158.777	Yes	2158.806	No	N/A
Hex6dHex1HexNAc5	2174.772	Yes	2174.768	Yes	2174.807
Hex5dHex1HexNAc6	2215.798	Yes	2215.847	Yes	2215.837
Hex6HexNAc6	2231.973	No	N/A	Yes	2231.828
Hex6HexNAc5NeuAc1	2319.809	Yes	2319.804	Yes	2301.869 ^a^
			2341.804 ^e^		2347.878 ^b^
Hex6dHex2HexNAc5	2320.829	Yes	2320.746	Yes	2320.902
Hex6dHex1HexNAc6	2377.851	Yes	2377.859	Yes	2377.897
Hex7HexNAc6	2393.846	Yes	2393.796	Yes	2393.888
Hex6dHex1HexNAc5NeuAc1	2465.867	Yes	2465.803	Yes	2447.904 ^a^
			2487.886 ^e^		2493.916 ^b^
Hex6dHex3HexNAc5	2466.887	Yes	2466.716	No	N/A
Hex7dHex1HexNAc6	2539.904	Yes	2539.856	Yes	2539.886
Hex7dHex2HexNAc6	2685.962	Yes	2686.010	Yes	2686.028
Hex6dHex1HexNAc5NeuAc2	2756.962	Yes	2756.976	Yes	2767.056 ^c^
			2778.961 ^e^		2813.066 ^d^
Hex8HexNAc7	2758.978	Yes	2758.976	No	N/A

^a^ Glycan with lactonized sialic acid; ^b^ Glycan with esterified sialic acid; ^c^ Glycan with one lactonized sialic acid and one esterified sialic acid; ^d^ Glycan with two esterified sialic acids; ^e^ Native glycan two sodium adducts.

### 3.2. Similarities of Glycan Distribution across Tissue Types

**Figure 3:**
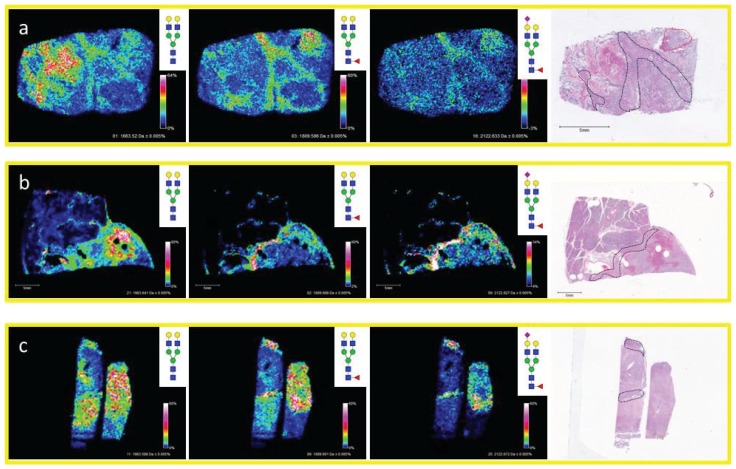
Diverse Organs with Similar Properties Display Conserved Glycan Patterns. Fibrous tissue from three diverse tissue types were analyzed by MALDI-IMS and shared similar *N*-glycan properties. Shown are images from lymph tissue with a ccRCC metastasis (**a**); pancreatic cancer tissue with complex histopathology (**b**); and liver tissue with complex histopathology (**c**). In each tissue, the ions corresponding to Hex5HexNAc4 (*m*/*z* = 1663.582 + Na), Hex5dHex1HexNAc4 (*m*/*z* = 1809.661 + Na), and Hex5dHex1HexNAc4NeuAc1 (*m*/*z* = 2122.720 + 2Na) display similar localization patterns with each being elevated in the fibrous tissue regions. Fibroconnective tissue regions are outlined in black on the H & Es while fibrotic ccRCC tumor tissue is outlined in red. The core fucosylation in the indicated structures has yet to be confirmed by other methods.

The distribution of *N*-glycans in different fibroconnective tissues from other sources besides HCC tissues was further assessed. Lymph tissue with a clear cell renal cell carcinoma (ccRCC) metastasis (Figure 3a), pancreatic cancer and adjacent normal tissue (Figure 3b), and the hepatocellular carcinoma tissue presented earlier (Figure 3c), all with regions of fibrous and/or fibroconnective tissue, were analyzed by MALDI-IMS for *N*-glycans. The regions of fibroconnective tissue (outlined in black) and fibrous tumor tissue (outlined in red) are defined on the H & E image for each tissue (Figure 3). In each tissue, three diantennary *N*-glycans were commonly detected at higher levels in the fibrous/fibroconnective tissue compared to adjacent regions: Hex5HexNAc4 (*m*/*z* 1663.582), Hex5dHex1HexNAc4 (*m*/*z* 1809.661), and Hex5dHex1HexNAc4NeuAc1 (*m*/*z* 2122.720; [M − H + 2Na]^+^). In all tissues, the Hex5dHex1HexNAc4NeuAc1 glycan had the highest specificity for fibrous tissue regions. The Hex5HexNAc4 was detected at greater signal intensity in more regions of the tissues, and we have also noticed that this glycan is likely a marker of tissue regions where blood is present, illustrated in Figure 3b. These same glycans have also been detected primarily in stroma regions of prostate cancer tissues [28]. We hypothesize this shared glycan structural motif across tissues reflects the glycoproteins carrying them, most likely collagen and collagen-binding proteins. Glycoproteomic experiments are ongoing to assess this.

### 3.3. Validation of N-Glycans Structures by off-Slide Glycomic Analysis

**Figure 4:**
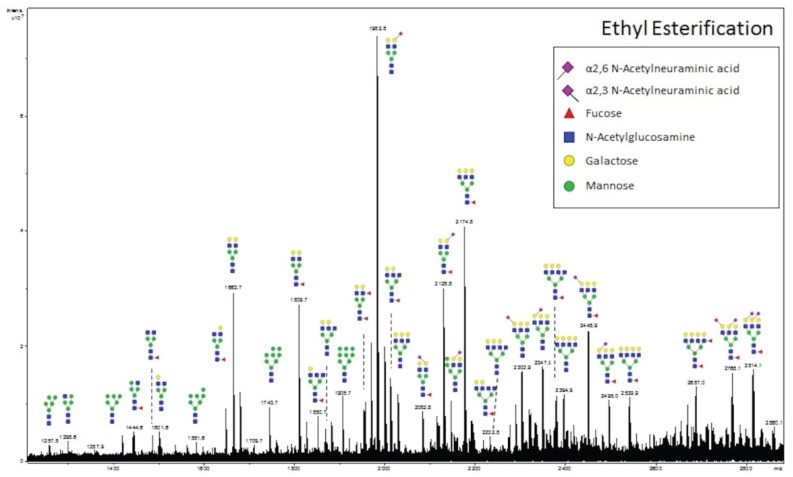
Off-Tissue Ethyl Esterification of HCC Glycans Correlates with MALDI-IMS Results. *N*-glycans extracted from HCC tissue sections were modified by ethyl esterification followed by cotton HILIC tip enrichment [22,26]. Enriched glycans were spotted on a MALDI plate and analyzed in positive ion mode by MALDI-FTICR. The core fucosylation in the indicated structures has yet to be confirmed by other methods.

MALDI ionization of glycans often results in the loss of labile sialic acids from glycans. We performed a recently introduced glycan modification approach, termed ethyl esterification [22], to assess whether the loss of sialic acids is occurring in MALDI-IMS experiments. This procedure results in lactonization of α-2,3 linked sialic acids and ethyl esterification of α-2,6 linked sialic acids, resulting in a 46.04 Da mass shift [22]. Following ethyl esterification of glycans, glycans were enriched using cotton-HILIC tips. Two HCC slides from adjacent slices were prepared for on-slide PNGaseF digestion. One slide was processed as described for MALDI-IMS analysis. For the other slide, glycans were retrieved from the tissue, subjected to the ethyl esterification protocol, and spotted on an anchorchip MALDI plate. An annotated spectrum is provided (Figure 4). A list of 33 representative *N*-glycans present in the MALDI-IMS and/or ethyl esterification experiments, as well as the glycan’s theoretical *m*/*z* value is provided in Table 1. The results demonstrate a close overlap of observed *m*/*z* values for both approaches with theoretical *m*/*z* values obtained from GlycoWorkbench. Additionally, a majority of *N*-glycans were present in both the MALDI-IMS and off-tissue ethyl esterification experiments. However, when comparing the ethyl esterification average spectrum (Figure 4) with the MALDI-IMS average spectrum (Figure 1a), it is evident that the peak intensities of complex and sialylated *N*-glycans are more predominant in the ethyl esterification spectrum compared to the MALDI-IMS experiment: an example being Hex5HexNAc4NeuAc1 which has the highest signal intensity in the ethyl esterification spectrum (*m*/*z* 1936.692 and *m*/*z* 1982.733), but is only detected as minor peaks in the imaging average spectrum (*m*/*z* 1954.589 and *m*/*z* 1976.707). This result may be attributed to a reduction in signal intensity of sialylated *N*-glycans in MALDI-IMS due to a loss of sialic acid upon in-source decay, reflected in the comparatively higher level of detection of Hex5HexNAc4 (*m*/*z* 1663.582) in the MALDI-IMS average spectrum. However, as this comparison involves a purified glycan preparation *vs*. the analysis of glycans directly from the more complex tissue without any purification, the differences between the two may represent other conditions besides in-source decay.

**Figure 5:**
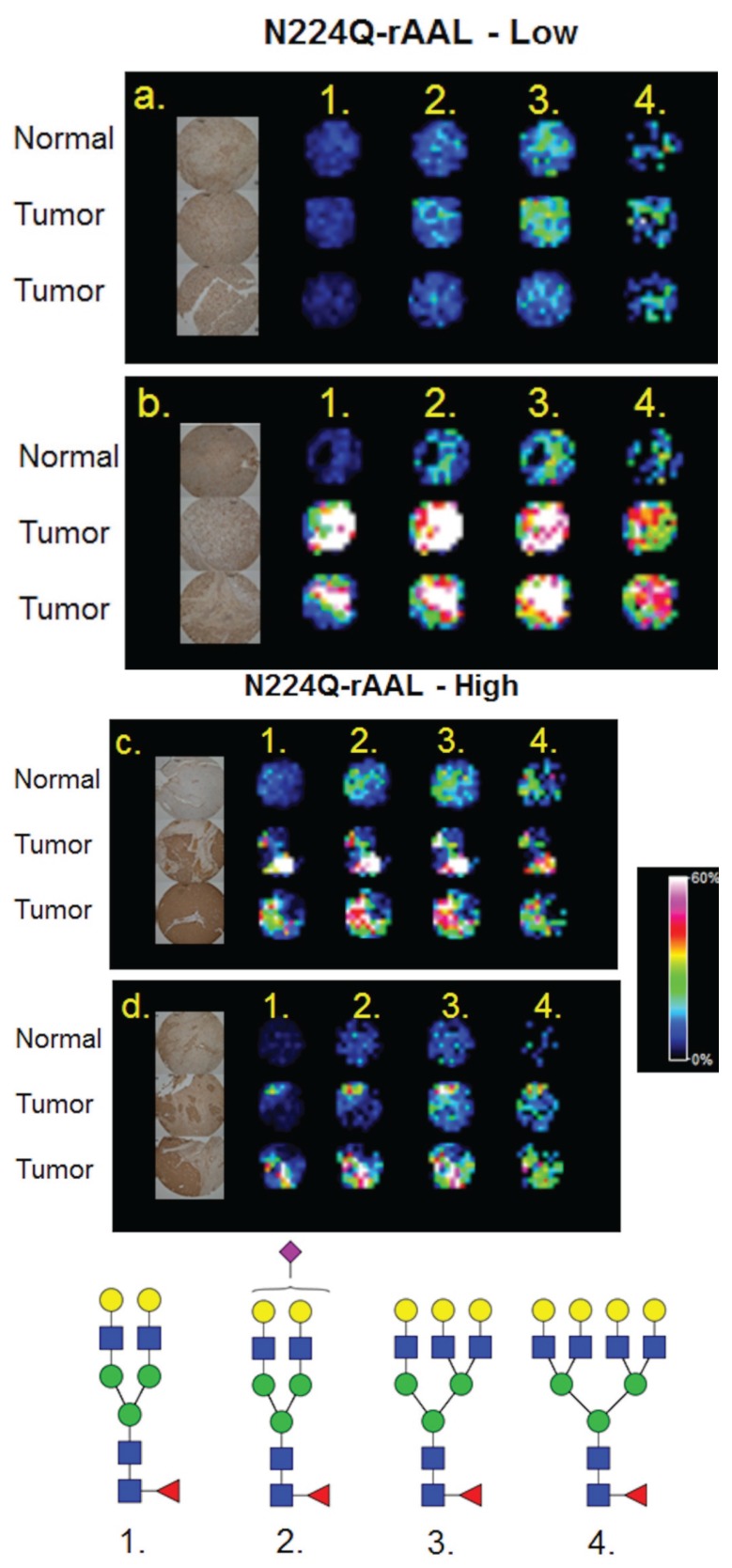
Comparison of Core Fucose Glycans Detected by Lectin Histochemistry and MALDI-IMS. A commercial HCC TMA (Biochain) containing 16 sets of tumor (2 cores each), and matched non-tumor adjacent (one core) tissues was profiled by MALDI-IMS [21], or stained by lectin histochemistry with a recombinant core fucose binding lectin, N224Q-rAAL [27]. Four representative TMA core sections were selected for comparison. Lectin histochemistry indicated tumor cores with low (top panel, (**a**,**b**)) or high (bottom panel, (**c**,**d**)) staining. Additional staining images are provided in Supplemental Figure S2. The image for each lectin stained core was captured individually, and images in the panel are from a 4× magnification by light microscopy. The individual core images were re-grouped in the panel to match the MALDI-IMS image orientation. Shown in the MALDI images are four mono-fucosylated glycans: 1. Hex5dHex1HexNAc4 (*m*/*z* = 1809.661); 2. Hex5dHex1HexNAc4NeuAc1 (*m*/*z* = 2122.720 + 2Na); 3. Hex6dHex1HexNAc5 (*m*/*z* = 2174.772); 4. Hex7dHex1HexNAc6 (*m*/*z* = 2539.904).

### 3.4. Detection of Core Fucosylation in HCC Tissue Microarrays

In addition to the dissociation of labile residues, another historical structural limitation of MALDI analysis is differentiating glycans with a core fucose *vs*. those with an outer arm fucose. To explore solutions for this limitation, the localization of glycans from a MALDI-IMS experiment were compared to a histochemistry staining with a core fucose binding lectin. We have recently reported MALDI-IMS profiling can be applied to TMA, and demonstrated this feature on a small HCC TMA of 48 tissue cores [22]. A recently described recombinant lectin [27], N224Q-rAAL, that is specific to binding of core fucosylated glycans was used to stain a serial section of the HCC TMA. This lectin has been used to detect increased core fucosylation of *N*-glycans associated with HCC [27]. Staining intensities of high *vs*. low core fucose expressing tissues were determined and compared to the corresponding MALDI glycan images of compositions with single fucoses. As shown in Figure 5 for a subset of tissue cores with low and high core fucose staining, there was a consistent detection of four singly fucosylated glycans by MALDI imaging in tumor cores. These glycans display a higher intensity in tissue cores that exhibit high levels of lectin staining (Figure 5c,d). Conversely, another tissue core set with low N224Q-rAAL staining had significantly lower signal intensities of the same four glycans (Figure 5a). In contrast, a different low N224Q-rAAL staining core set had high amounts of signal intensities for the mono-fucosylated glycans, suggesting that the fucose position in these glycans could be an outer arm modification (Figure 5b). Higher resolution light field microscope images of the lectin staining of these tissue cores are shown in Supplemental Figure S2. This example illustrates the potential utility of combining class specific lectin staining of tissues with the MALDI-IMS detection of individual glycans, an approach particularly amenable to fucose and sialic acid containing complex *N*-glycans.

**Figure 6:**
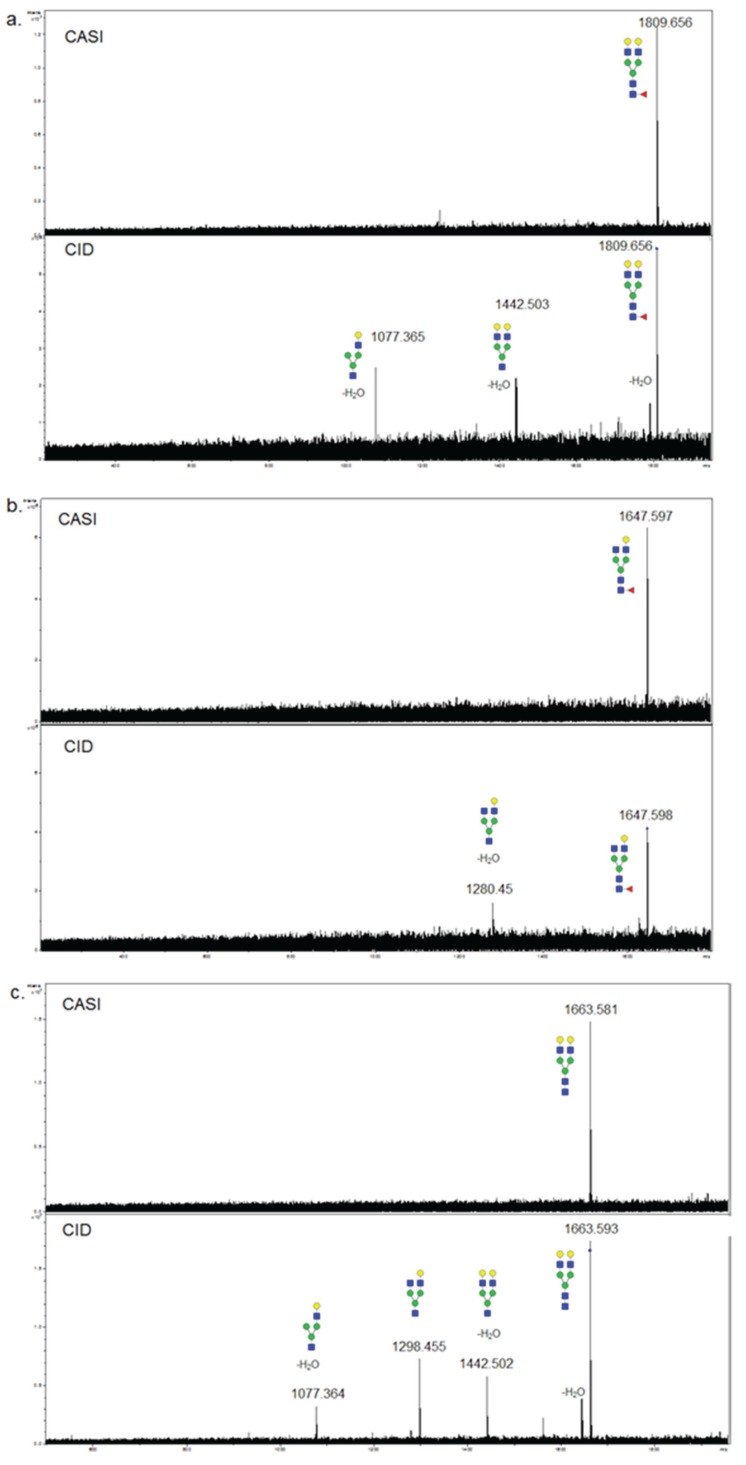
Collision-Induced Disocciation of Singly Fucosylated Tissue *N*-glycans. *N*-glycans were fragmented by CASI-CID directly from tissue sections as previously described [20,21]. The top panel for each glycan is the spectra following CASI capture, and the bottom panels show the fragmentation ions after CID; (**a**) Hex5dHex1HexNAc4 (*m*/*z* = 1809.656); (**b**) Hex4dHex1HexNAc4 (*m*/*z* = 1647.598); (**c**) Hex5HexNAc4 (*m*/*z* = 1663.581).

Multiple approaches will be needed to clarify the positions of the fucoses in these glycans, and will involve tandem mass spectrometry characterization. The FTICR-MALDI used in these studies is capable of selective ion capturing, termed CASI, and when coupled with collision-induced dissociation (CID), can determine glycan composition as previously reported [21]. However, these were proof-of-concept examples. To examine whether core fucosylation could be determined by this process, further analysis was done from single fucosylated glycans captured by CASI/CID directly from tissue. As shown in the spectra in Figure 6, CID of two singly fucosylated glycans of Hex5dHex1HexNAc4 (*m*/*z* = 1809.656; Figure 6a) and Hex4dHex1HexNAc4 (*m*/*z* = 1647.598; Figure 6b) resulted in loss of the terminal *N*-acetylglucosamine and fucose. The CID of a non-fucosylated Hex5HexNAc4 (*m*/*z* = 1663.581) is shown in comparison (Figure 6c). The peak isolated by CASI is shown in the top panel for each glycan. This general approach should be applicable to assist in distinguishing core fucosylation of simpler *N*-glycans detected by MALDI-IMS.

## 4. Discussion

Profiling of *N*-glycans in fresh/frozen tissues, FFPE tissue blocks and TMAs is a new application of MALDI-IMS methodology and new approach in glycobiology that has the potential to identify systemic disease markers and elucidate disease etiology. This methodology is particularly relevant for cancer tissues, as most known cancer biomarkers are glycoproteins or carbohydrate antigens [23,24], including AFP for HCC [2]. The MALDI-IMS glycan profiling data presented herein, although from only a limited number of HCC tissues, illustrates this potential. Compared to other biomolecules targeted by MALDI-IMS like lipids, metabolites and proteins/peptides, *N*-glycans offer a particular advantage in that there are relatively fewer total glycan ion signals that are detected in a given tissue. Assigning the underlying composition for each glycan is rather straight-forward on the basis of accurate masses, albeit anomeric linkages are not differentiated. In addition to mass accuracy, glycan compositions can be validated by a variety of approaches, such as CID and derivatization. Structural standards for many of the simpler glycans are available, several glycan reference databases are available, and CID of glycans directly from tissue slides is possible using the MALDI-FTICR instrument [21].

Knowing the identity of the glycan offers many other opportunities for application to cell biology and genomic studies, particularly as these structures are synthesized by specific glycosyltransferases, and modified by specific glycosidases. This facilitates comparative analysis of genomic data for these enzymes from the same tissues, and several studies have been reported attempting to develop predictive algorithms for MALDI-detected glycans and gene transcripts [29,30,31]. Knowing the glycan composition also allows inference of different cell biology aspects related to biosynthesis, vesicular trafficking or degradation processes involving *N*-glycans. We have found across multiple tumor types, and illustrated herein, that identifying a specific tumor glycan profile in tissues is fairly straightforward [21,28]. Herein, this principle has been further expanded as consistent glycan signatures for fibroconnective tissue have been observed across cancer types (Figure 3). We are further testing whether detecting changes in glycan profiles can be used as surrogate indicators of changes in glycoprotein expression associated with cancer development and progression, as well as responses to treatment. Current efforts are also directed at using the *N*-glycan profiles as a tissue map to identify disease-specific regions of interest for subsequent glycopeptide analysis strategies [32,33].

There still remain many challenges associated with improving the approach, some of which were highlighted in the results. Detection of high mass *N*-glycans by MALDI mass spectrometry, particularly those glycans containing multiple sialic acids, has always been a challenge as previously described [34,35,36,37]. We are addressing these challenges in three ways, at the instrumentation level, with on-slide analysis approaches, and with off-slide analysis approaches. The source configuration of the Solarix 7T FTICR instrument used for these experiments is already inherently amenable to better detection of sialic acid-containing glycans. Compared to most MALDI-TOF instruments (*i*.*e*., non-FTICR), less vacuum and a cooling gas following laser desorption/ionization is present. This combination has been reported to aid in the retention of sialic acids on glycoconjugates [38,39]. The ability to selectively capture specific glycan ions directly from tissue for CID is also an advantage of the Solarix instrument [21]. Comparison of *N*-glycan profiles of the same tissue slides in different MALDI-TOF instruments is ongoing, with emphasis on how detection of sialic acid containing glycans differ across instrument configurations. In this context, different MALDI matrix formulations are also being tested and compared. Combining these approaches with glycan class-specific lectin staining is another complementary approach, highlighted in Figure 5. These are all cumulatively being assessed in the optimization for detection of higher mass glycan species.

Currently, based on mass accuracy of standards and derivatization experiments [20,21], we show that while sialylated *N*-glycans are detected in MALDI-IMS (Figure 1a), they appear to be of lower abundance than may be naturally occurring (Figure 4). Attempts to globally modify *N*-glycan reducing ends (*i*.*e*., 3-aminoquinoline, [40]) or specifically modifying labile sialic acid residues (*i*.*e*., p-toluidine or ethyl esterification) represent potential solutions, as previously reported [22,41,42]. We believe that some modification of the above off-slide derivation approaches used for *N*-glycans could be adapted to an on-slide MALDI-IMS tissue profiling workflow. Additionally, performing MALDI-IMS of *N*-glycans in negative ion mode could be a reasonable approach for partially retaining sialic acids of *N*-glycans, although much optimization remains to be done. For both fucose and sialic acid residue determination, the use of linkage specific fucosidases and sialidases are additional options. Applying sialidase in combination with PNGaseF followed by MALDI-IMS glycan imaging has been demonstrated for mouse brain tissues [20].

Given the ability to use FFPE tissues as the primary source for *N*-glycan imaging and their general availability, there is a potential to leverage this approach to discover glycan biomarkers of HCC and other cancer types. To facilitate biomarker discovery, the described techniques must be used with large numbers of tissue samples on sample slides or in tissue microarray format, highlighting the need for an accurate method to directly compare between samples/experiments. In the examples provided for HCC, *N*-glycan signatures were capable of distinguishing tumor from non-tumor tissue regions. To determine the significance of these glycans, a similar study involving an increased number of tissue cores is required. Ongoing research is directed at determining best practices for data normalization and incorporating internal standards into experimental workflows, and are issues common to all MALDI-IMS experiments [43]. Only after large enough data sets are generated will we be able to determine the best approach to analyze these larger sample sets. Use of TMAs for glycan MALDI-IMS can certainly facilitate the increase in sample numbers [44]. Use of MALDI-IMS alone may not be enough, as it is the type of method that can be complemented with existing and emerging quantitative mass spectrometry approaches [45], as well as lectin staining of tissues directly or use of lectin arrays [46]. A recent report using mouse kidney tissues has linked MALDI-IMS of *N*-glycans with LC-MS/MS characterization of the glycans [47]. This combination of MALDI, LC-MS/MS and array approaches will likely yield a more complete analysis option of FFPE tumor tissues.

## 5. Conclusions

The profiling and tissue mapping of *N*-glycans offers many new avenues of glycobiology research that can be initiated by, and complemented with, MALDI-IMS strategies. Challenges and limitations of the approach are evident, but we believe most can be addressed moving forward as more samples are analyzed, new derivation and matrix strategies tested, and through long-term, continued improvement in instrumentation platforms.

## Supplementary Files

Supplementary File 1
