# Supplementary material for: Two-Dimensional N-Glycan Distribution Mapping of Hepatocellular Carcinoma Tissues by MALDI-Imaging Mass Spectrometry
Source: Biomolecules. 2015 Oct 15;5(4):2554–72. doi: 10.3390/biom5042554 (PMC4693247; doi:10.3390/biom5042554)
Supplement: Supplementary File 1 [file biomolecules-05-02554-s001.pdf]

Supplementary Information

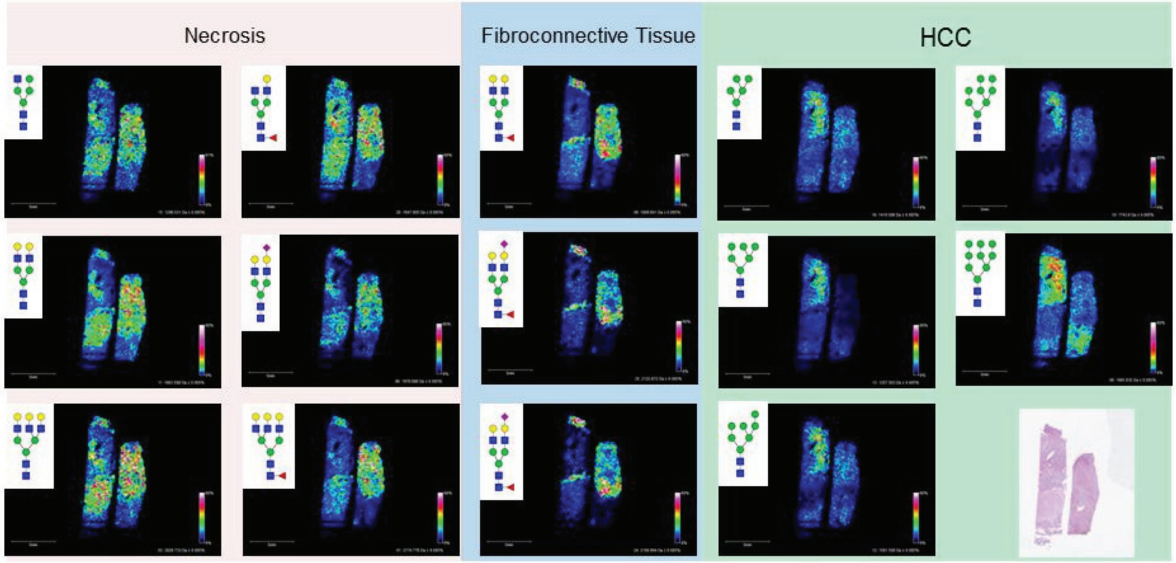

Figure S1. *N*-glycans Can Distinguish Complex Histopathology in HCC Tissue. The same HCC tissue shown in Figure 1 was further evaluated for glycan distribution patterns associated with histopathology features. Glycan distribution followed three primary patterns, as demonstrated in the image panel. Later histopathology analysis characterized these regions as hepatocellular carcinoma (green background), necrosis (pink background), and fibroconnective tissue (blue background). An overlay of one glycan from each region is provided (a). An annotated H & E of this tissue section is provided in Figure 1.

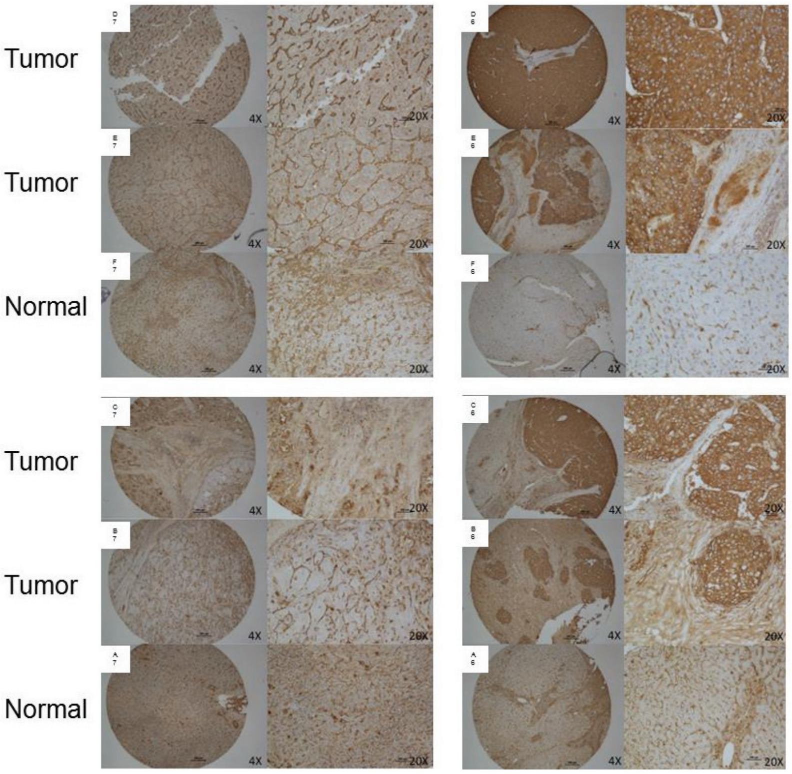

Figure S2. N224Q-rAAL Histochemistry of HCC TMA-4× and 20× Magnifications.
